# Intermediate Levels of *Bacillus subtilis* CodY Activity Are Required for Derepression of the Branched-Chain Amino Acid Permease, BraB

**DOI:** 10.1371/journal.pgen.1005600

**Published:** 2015-10-16

**Authors:** Boris R. Belitsky, Shaun R. Brinsmade, Abraham L. Sonenshein

**Affiliations:** Department of Molecular Biology and Microbiology, Tufts University School of Medicine, Boston, Massachusetts, United States of America; Max Planck Institute for Terrestrial Microbiology, GERMANY

## Abstract

The global transcriptional regulator, CodY, binds strongly to the regulatory region of the *braB* gene, which encodes a *Bacillus subtilis* branched-chain amino acid (BCAA) permease. However, under conditions that maximize CodY activity, *braB* expression was similar in wild-type and *codY* null mutant cells. Nonetheless, expression from the *braB* promoter was significantly elevated in cells containing partially active mutant versions of CodY or in wild-type cells under growth conditions leading to intermediate levels of CodY activity. This novel pattern of regulation was shown to be due to two opposing mechanisms, negative and positive, by which CodY affects *braB* expression. A strong CodY-binding site located downstream of the transcription start point conferred negative regulation by direct interaction with CodY. Additionally, sequences upstream and downstream of the promoter were required for repression by a second pleiotropic *B*. *subtilis* regulator, ScoC, whose own expression is repressed by CodY. ScoC-mediated repression of *braB* in *codY* null mutants cells was as efficient as direct, CodY-mediated repression in wild-type cells under conditions of high CodY activity. However, under conditions of reduced CodY activity, CodY-mediated repression was relieved to a greater extent than ScoC-mediated repression was increased, leading to elevated *braB* expression. We conclude that restricting increased expression of *braB* to conditions of moderate nutrient limitation is the raison d’être of the feed-forward regulatory loop formed by CodY and ScoC at the *braB* promoter. The increase in BraB expression only at intermediate activities of CodY may facilitate the uptake of BCAA when they are not in excess but prevent unneeded BraB synthesis when other BCAA transporters are active.

## Author Summary

Expression of *Bacillus subtilis* BraB, a branched-chain amino acid permease, is under both negative and positive control by a global transcriptional regulator CodY. The negative control is direct and the positive control is indirect and mediated by another *B*. *subtilis* pleiotropic transcriptional regulator, ScoC, which, in turn, is repressed by CodY. Thus, CodY and ScoC form a feed-forward regulatory loop at the *braB* promoter. In a very unusual manner, the interaction of CodY and ScoC results in high *braB* expression only at intermediate CodY activities; *braB* expression remains low both at high and low CodY activities. The novel regulation of *braB* shows that important, novel regulatory phenomena can be missed by analyzing null mutants in regulatory genes but revealed by using mutants with partial activity.

## Introduction

BraB is one of three permeases demonstrated to be involved in the uptake of branched-chain amino acids (BCAA) in *Bacillus subtilis* [1]. Given the important role of BCAA in cell metabolism, it is not surprising that the synthesis of the permeases is strictly regulated and coordinated. The most efficient BCAA permease, BcaP, is subject to very strong transcriptional repression by CodY [2], a global regulator in *B*. *subtilis* and other Gram-positive bacteria [3, 4]. A second permease, BrnQ, is subject to strong repression by AzlB, a member of the AsnC/Lrp family of transcriptional regulators, in response to an as yet unidentified signal [5]. The regulation of BraB synthesis has not been previously determined.

A fragment containing the regulatory region between the divergently transcribed *iscSB* (formerly *nifZ*) and *braB* genes was found to bind CodY strongly *in vivo* in a ChIP-to-chip experiment [6]. Moreover, a strong CodY-binding site in the *iscSB-braB* intergenic region was also detected *in vitro* during the global characterization of CodY-binding sites by IDAP-Seq [7]. The latter site is well-placed to serve as a potential site of regulation of *braB*. However, transcription of neither *braB* nor *iscSB* was altered **>**2.0-fold by a null mutation in *codY*, as detected in DNA microarray or RNA-Seq experiments [6, 8](http://www.genome.jp/kegg/expression/).

CodY controls directly or indirectly the transcription of more than 200 *B*. *subtilis* genes [7, 8]. The DNA-binding affinity of CodY from *B*. *subtilis* and many other species is increased by interaction with two types of ligands, the BCAA [isoleucine, leucine, and valine (ILV)] [9–11] and GTP [6, 11–14]. Thus, given the presence of CodY-binding sites in the putative *braB* regulatory region and the influence of BCAA on CodY activity, it was surprising that expression of *braB* was only minimally affected by a *codY* null mutation. We describe here a detailed analysis of the mechanisms by which CodY regulates *braB*.

The *braB* gene proved to be directly repressed by two proteins, CodY and another pleiotropic regulator, ScoC *(*formerly known as *hpr* or *catA)* [15–18]. Because CodY also represses *scoC* [19], CodY and ScoC form a feed-forward regulatory loop [20, 21] in which CodY acts an indirect positive regulator of *braB*. The opposing effects of fully active CodY balance each other; as a result, *braB* derepression could only be observed at intermediate levels of CodY activity or when both regulators are inactive. These findings emphasize that the phenotypes caused by null mutations in global regulatory protein genes can be misleading.

## Results

### *braB* CodY-binding sites

**Fig 1:**
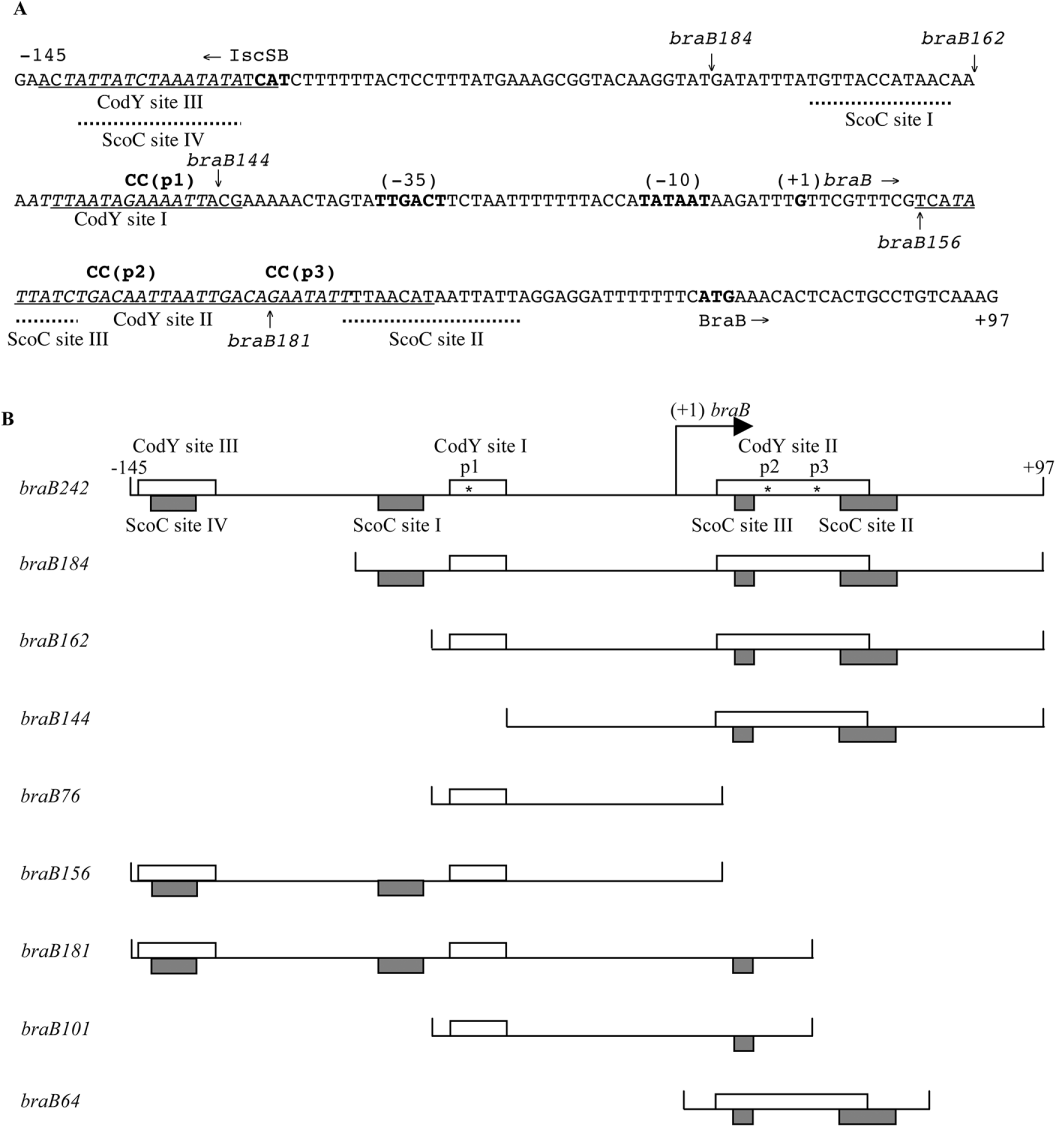
The sequence of the *braB* regulatory region and map of the promoter fragments used. A. The sequence (5’ to 3’) of the coding (non-template) strand of the *braB* regulatory region within the *braB242-lacZ* fusion. Coordinates are reported with respect to the transcription start point. The upstream boundary of the *braB184*, *braB162*, and *braB144* fusions at positions –87, -65, and -47, respectively, are indicated by vertical arrows above the sequence. The vertical arrows below the sequence indicate the junction points, at position +11 and +36, between the *braB* and *lacZ* sequences. The likely translation initiation codon, -10 and -35 promoter regions, and apparent transcription start point are in boldface. The directions of transcription and translation are indicated by the horizontal arrows. The sequences on the template strand that were protected by CodY or ScoC in DNase I footprinting experiments are underlined or shown by dotted horizontal lines below the sequence, respectively. The sequences of CodY-binding motifs are italicized and shown in Table 1. The mutated nucleotides are shown in lowercase above the sequence. B. Schematic maps of the *braB* fragments used to construct *lacZ* fusions or in DNA-binding experiments. The coordinates indicate the boundaries of different fusions with respect to the *braB* transcription start point. The location of the apparent transcription start point is indicated by the bent arrow. CodY- and ScoC-binding sites determined in DNase I footprinting experiments are shown as clear or shaded rectangles, respectively.

The unexpected absence of an effect of a *codY* null mutation on expression of a gene with a strong CodY-binding site in its putative regulatory region led us to analyze *braB* transcription in more detail. A primer extension experiment established that the 5’ end of the *braB* mRNA is located 72 bp upstream of the initiation codon. The sequences TTGACT and TATAAT, with one and no mismatches to the –35 and –10 regions of σ^A^-dependent promoters, respectively, and a 16-bp spacer region, can be identified upstream of the 5’ end location, suggesting that this position does in fact correspond to the transcription start point (Fig 1A). (Since *B*. *subtilis* σ^A^-dependent promoters rarely have a 16-bp spacer, our assignment of the -10 and -35 regions may be off by 1 or 2 bp.) A mutation, T(-29)C, located immediately downstream of the likely -35 region, reduced expression of a *braB-lacZ* fusion 6-fold (1.97±0.35 Miller units, see below), consistent with our assignment of the promoter.

**Fig 2:**
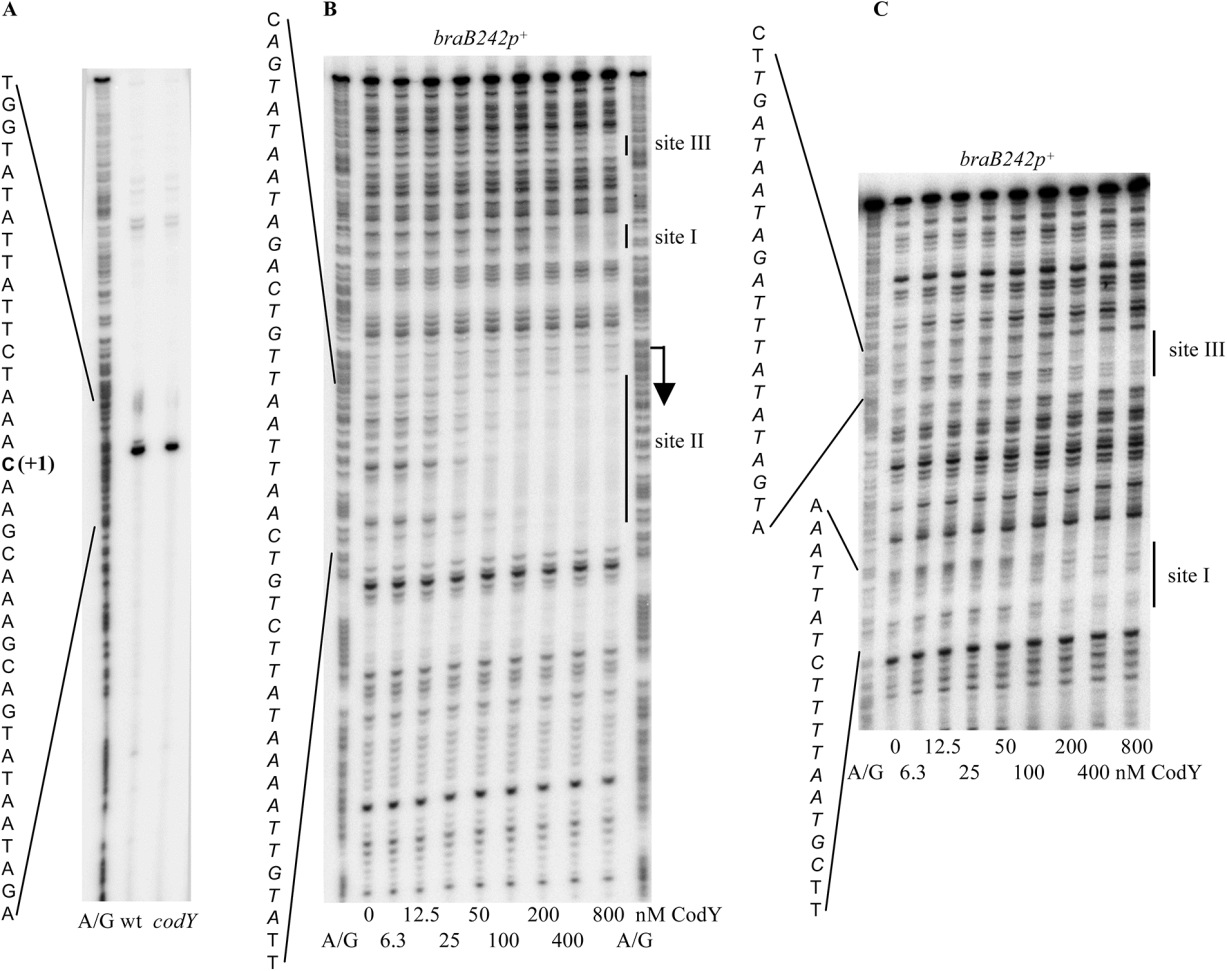
Determination of the *braB* transcription start point and CodY-binding regions. A. Primer extension analysis of the *braB* mRNA. Primer oBB102 annealing to the *lacZ* gene of the *braB242-lacZ* fusion was extended with reverse transcriptase using as the template total RNA from fusion-containing strains BB3076 (wt) and BB3079 (*codY*) grown in TSS + 16 aa medium. The A+G sequence of the template strand of pBB1593 determined from reactions primed with oBB102 is shown to the left. The apparent transcription start site of the *braB* gene is in bold and marked by the +1 notation. B. DNase I footprinting analysis of CodY binding to the *braB* regulatory region. The *braB242p* ^*+*^ DNA fragment obtained by PCR with oligonucleotides oBB67 and oBB102 and labelled on the template strand was incubated with increasing amounts of purified CodY in the presence of 10 mM ILV and 2 mM GTP and then with DNase I. The protected areas are indicated by vertical lines and the corresponding sequences are reported; the protected nucleotides are italicized. The apparent transcription start site and direction of transcription are shown by a bent arrow. CodY concentrations used (nM of monomer) are indicated below each lane. The A + G sequencing ladder of the template DNA strand is shown in the flanking lanes. C. Same as B, the gel was run longer to improve the resolution of the upstream CodY-binding sites III and I.

DNase I footprinting experiments showed that CodY protected two sites, I and II, within the 194-bp *iscSB-braB* intergenic region from positions -62 to -47 and +11 to +50 of the template DNA strand with respect to the *braB* transcription start point, respectively (Figs 1A, 2B and 2C). Site II is much stronger than site I. Binding to an additional very weak site, III, from positions –143 to –124, which is within the upstream *iscSB* gene, was observed only at high concentrations of CodY (≥200 nM) (Fig 2B and 2C).

The results of the footprinting experiments are consistent with the identification of a strong CodY-binding site in this area by ChIP-to-chip experiments [6]. Moreover, they confirmed and extended the results of the *in vitro* IDAP-Seq experiments, which identified a strong core binding site from positions +29 to +43, a much weaker core site, which ends at position -45, and an additional, very weak core site, ending at position -116 and detected only at a very high CodY concentration (1 μM) (core sites only include positions that are essential for CodY binding; the beginning positions of the two upstream core sites could not be determined due to limitations of the IDAP-Seq procedure) [7].

The *braB* regulatory region contains five 15-bp motifs, which resemble the 15-bp CodY-binding consensus sequence, AATTTTCWGAAAATT [22–24] (we use the terms “site” and “motif” to describe an experimentally determined location of CodY binding and a 15-bp sequence that is similar to the consensus motif, respectively). Site I of the *braB* gene overlaps CodY-binding motif 1, located between positions -64 and -50, that has 4 mismatches with respect to the CodY-binding consensus (Fig 1A and Table 1). The strong site II overlaps two adjoining versions of the 15-bp sequence, motifs 2 and 3, located between positions +14 and +43, each of which has three mismatches with respect to the consensus motif. Another 15-bp sequence, motif 4, with four mismatches is located from positions +40 to +54 and overlaps motif 3 by 4 bp. Site III overlaps CodY-binding motif 5, with 5 mismatches, located from positions -141 to -127 (Fig 1A and Table 1).

**Table 1:** CodY-binding motifs of the *braB* gene.

Motif	Sequence^a^	Number of mismatches	Location with respect to the transcription start point
Consensus	AATTTTCWGAAAATT	0	
*braB* 1	AtTTaatAGAAAATT	4	-64 to -50
*braB* 2	tATTaTCTGAcAATT	3	+14 to +28
*braB* 3^b^	AATTgaCAGAAtATT	3	+29 to +43
*braB* 4^b^	tATTTTaAcAtAATT	4	+40 to +54
*braB* 5	tATTaTCTaAAtATa	5	-141 to -127
*braB* 1 p1	AtTTaatA**cc**AAATT	6	-64 to -50
*braB* 2 p2	tATTaTCT**cc**cAATT	5	+14 to +28
*braB* 3 p3	AATTgaCA**cc**AtATT	5	+29 to +43

^*a*^Mismatches to the CodY-binding consensus motif are indicated by lower case letters. Mutations are in boldface.

^*b*^Parts of motifs 3 and 4 that overlap are underlined.

**Fig 3:**
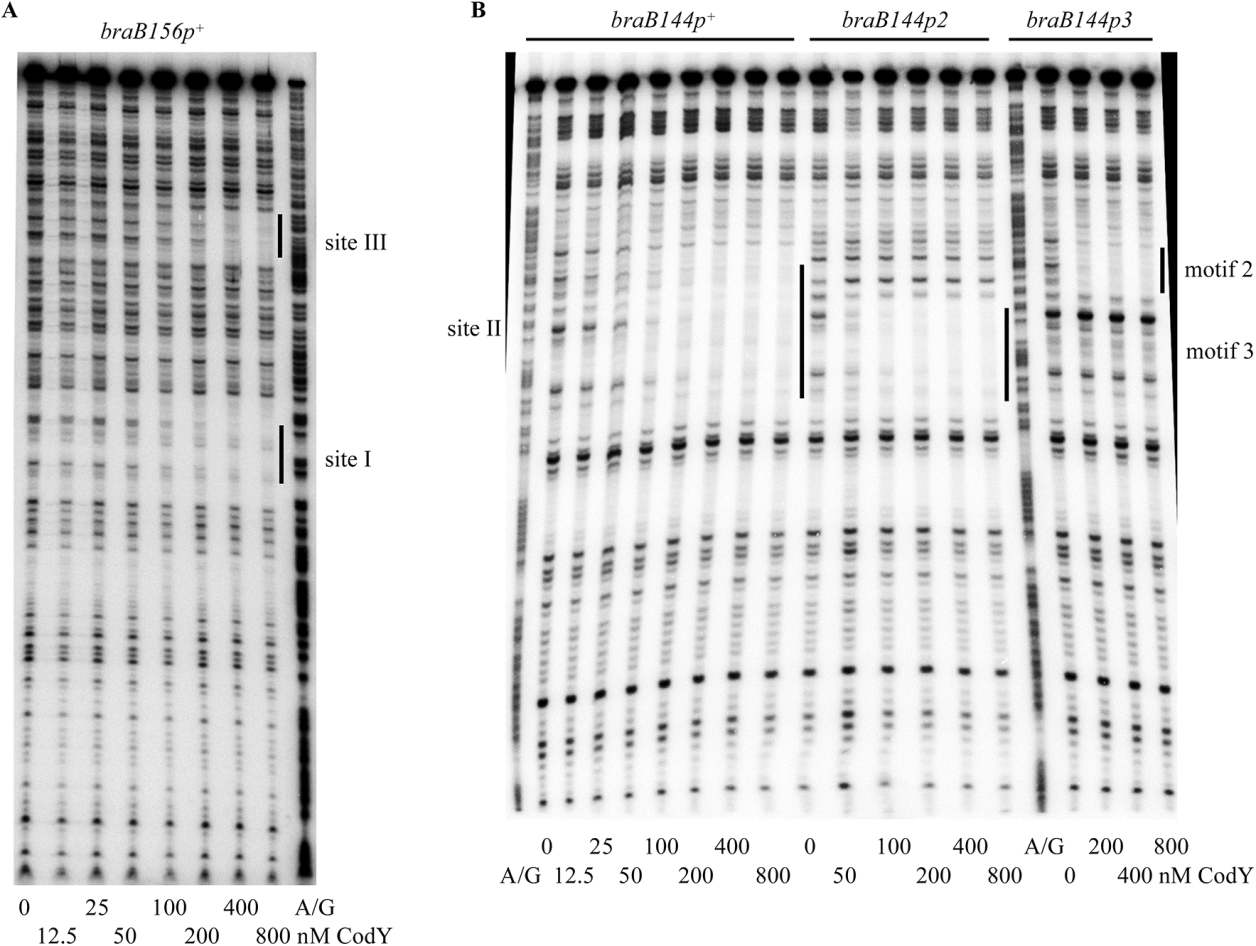
Role of *braB* CodY-binding sites in CodY binding. The *braB156p* ^*+*^ (A) or *braB144p* ^*+*^, *braB144p2*, and *braB144p3* (B) DNA fragments obtained by PCR with oligonucleotides oBB67 and oBB102 and labelled on the template strand were incubated with increasing amounts of purified CodY in the presence of 10 mM ILV and then with DNase I. The protected areas are indicated by vertical lines. CodY concentrations used (nM of monomer) are indicated below each lane. The A + G sequencing ladder of the template DNA strand is shown in the flanking lanes.

Binding of CodY to upstream *braB* sites occurred independently of the presence of the downstream site and vice versa (Figs 1 and 3; see below for generation of the truncated fragments), similar to the case for other genes containing multiple CodY-binding sites within their regulatory regions [2, 25, 26].

**Fig 4:**
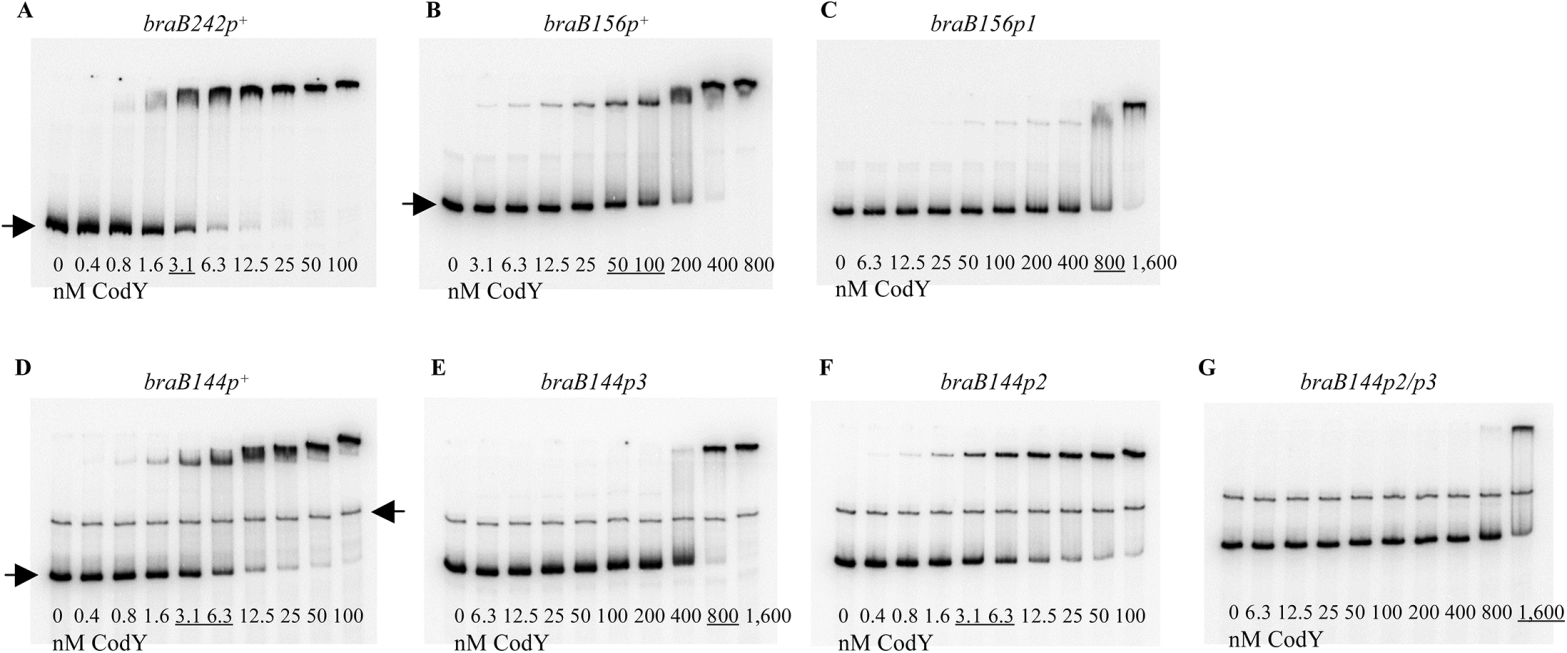
Gel shift assays of CodY binding to *braB* fragments. The *braB242p* ^*+*^ (A), *braB156p* ^*+*^ (B), *braB156p1* (C), *braB144p* ^*+*^ (D), *braB144p3* (E), *braB144p2* (F), and *braB144p2/3* (G) DNA fragments labeled on the template strand were incubated with increasing amounts of purified CodY in the presence of 10 mM ILV. The fragments were obtained by PCR with oligonucleotides oBB67 and oBB102 (A), oBB358 and oBB102 (B, C), and oBB422 and oBB102 (D-G) and their positions in the gel are indicated by right-pointing arrows. The unspecific DNA fragment, present in some panels, is indicated by a left-pointing arrow. CodY concentrations used (nM of monomer) are reported below each lane; concentrations corresponding to the apparent K_D_ for binding are underlined.

In gel-shift experiments, CodY bound to DNA fragments containing only sites III and I (*braB156*) or only site II (*braB144*) with apparent dissociation constants (K_D_) of ∼75 nM and ∼4 nM, respectively, compared with ∼3 nM for the full-length fragment, *braB242* (Fig 4A, 4B and 4D) (K_D_ reflects the CodY concentration needed to shift 50% of DNA fragments under conditions of vast CodY excess over DNA). Complexes with lower mobility were formed at higher concentrations of CodY for all fragments, indicating apparent changes in stoichiometry of CodY binding (Fig 4).

### Regulation of *braB* expression

We constructed a transcriptional fusion (*braB242-lacZ*) containing a 242-bp fragment that includes the entire *iscSB-braB* intergenic region (Fig 1). Under conditions of maximal CodY activity, in cells grown in TSS glucose–ammonium medium supplemented with ILV and a mixture of 13 other amino acids (referred to here as the 16 aa-containing medium), fusion expression in a *codY* null mutant strain was very similar (1.3-fold less) to that in the wild-type strain (Table 2, strains BB3076 and BB3079). Consistent with the *lacZ* fusion results, only very weak, positive regulation (1.6- to 1.9-fold) in amino acid-containing medium was detected in microarray or RNA-Seq experiments by comparing wild-type and *codY* null mutant strains [6, 8].

**Table 2:** CodY- and ScoC-mediated regulation of *lacZ* fusions.*^a^*

Strain	Fusion type	Regulatory sites within fusion	Relevant genotype	Addition to the medium	β-Galactosidase activity	*codY/codY* ^*+*^ fold regulation	*scoC/scoC* ^*+*^ fold regulation
BB3076	*braB242p* ^*+*^	CodY III, I, and II	wild type	16 aa	12.2±0.80	0.79	0.88
		ScoC I, II, and II					
BB3079		I	*codY*	16 aa	9.62±1.06		11.9
BB3847			*scoC*	16 aa	10.7±0.18	10.7	
BB3835			*codY scoC*	16 aa	114.2±4.40		
BB3076			wild type	13 aa	32.4±3.25	0.25	1.48
BB3979			*codY*	13 aa	8.13±0.20		11.7
BB3847			*scoC*	13 aa	48.0±3.45	2.0	
BB3835			*codY scoC*	13 aa	95.3±10.1		
BB3719	*braB184p* ^*+*^	CodY I and II	wild type	16 aa	16.6±0.60	1.1	0.95
		ScoC I, II and III					
BB3720			*codY*	16 aa	17.8±0.95		7.4
BB3857			*scoC*	16 aa	15.8±	1.31	8.3
BB3858			*codY scoC*	16 aa	130.9±15.8		
BB3811	*braB162p* ^*+*^	CodY I and II	wild type	16 aa	10.7±0.02	12.4	0.98
		ScoC II and III					
BB3816			*codY*	16 aa	132.9±0.95		0.77
BB3859			*scoC*	16 aa	10.5±0.90	9.8	
BB3860			*codY scoC*	16 aa	102.6±3.25		
BB3122	*braB144p* ^*+*^	CodY II	wild type	16 aa	6.80±0.07	12.6	0.79
		ScoC II and III					
BB3126			*codY*	16 aa	86.0±7.81		0.71
BB3891			*scoC*	16 aa	5.40±0.04	11.3	
BB3892			*codY scoC*	16 aa	60.8±7.40		
BB3123	*braB156p* ^*+*^	CodY III and I	wild type	16 aa	53.2±6.95	0.86	1.0
		ScoC I					
BB3127			*codY*	16 aa	45.8±7.25		0.86
BB3863			*scoC*	16 aa	53.9±3.40	0.73	
BB3864			*codY scoC*	16 aa	39.2±1.80		
BB3827	*braB181p* ^*+*^	CodY III and I	wild type	16 aa	372.2±0.75	0.85	1.0
		ScoC I and III					
BB3829			*codY*	16 aa	317.9±20.9		0.80
BB3861			*scoC*	16 aa	374.8±12.5	0.68	
BB3862			*codY scoC*	16 aa	253.2±34.3		
BB3821	*braB76p* ^*+*^	CodY III and I	wild type	16 aa	45.1±4.95	1.1	
		ScoC III					
BB3824			*codY*	16 aa	47.5±0.70		
BB3828	*braB101p* ^*+*^	CodY III and I	wild type	16 aa	305.0±38.5	0.88	
		ScoC III					
BB3830			*codY*	16 aa	267.9±15.5		

^*a*^Cells were grown in TSS glucose-ammonium medium with a mixture of 13 or 16 aa, as indicated. β-Galactosidase activity was assayed and expressed in Miller units ± standard error of the mean.

### *braB* expression is increased only at intermediate levels of CodY activity

The activity of CodY is reduced to intermediate levels when some amino acids are removed from the medium and decreases strongly in the absence of all amino acid supplements [2, 27]. Expression of the *braB242-lacZ* fusion in the wild-type strain in the absence of any amino acids or in the presence of ILV only was very similar to that in the presence of 16 aa (11.3 to 14.7 MU versus 12.2 MU). Unexpectedly, almost 3-fold higher activity was found in 13 aa-containing medium (i.e., in the absence of ILV), indicating that CodY, at an intermediate level of activity, may serve as a positive regulator of *braB* (Table 2).

To test whether expression of the *braB* gene indeed responds differentially to varying levels of CodY activity *in vivo* we made use of a previously constructed set of mutant forms of CodY that have different levels of residual responsiveness to ILV. Most of these proteins have alterations in amino acids that form the ILV-binding pocket; they are expressed at wild-type levels and have undiminished activity in effector-independent DNA binding [2, 8, 28]. Since the population of CodY molecules in the cell is in equilibrium between the liganded and unliganded forms of the protein, the unliganded fraction of the population of a mutant protein that has lower affinity for ILV will be greater than for the wild-type protein at a given intracellular ILV concentration. That is, a mutant strain containing a form of CodY that has low affinity for ILV behaves functionally equivalently to the wild-type strain that has a low intracellular pool of ILV.

**Fig 5:**
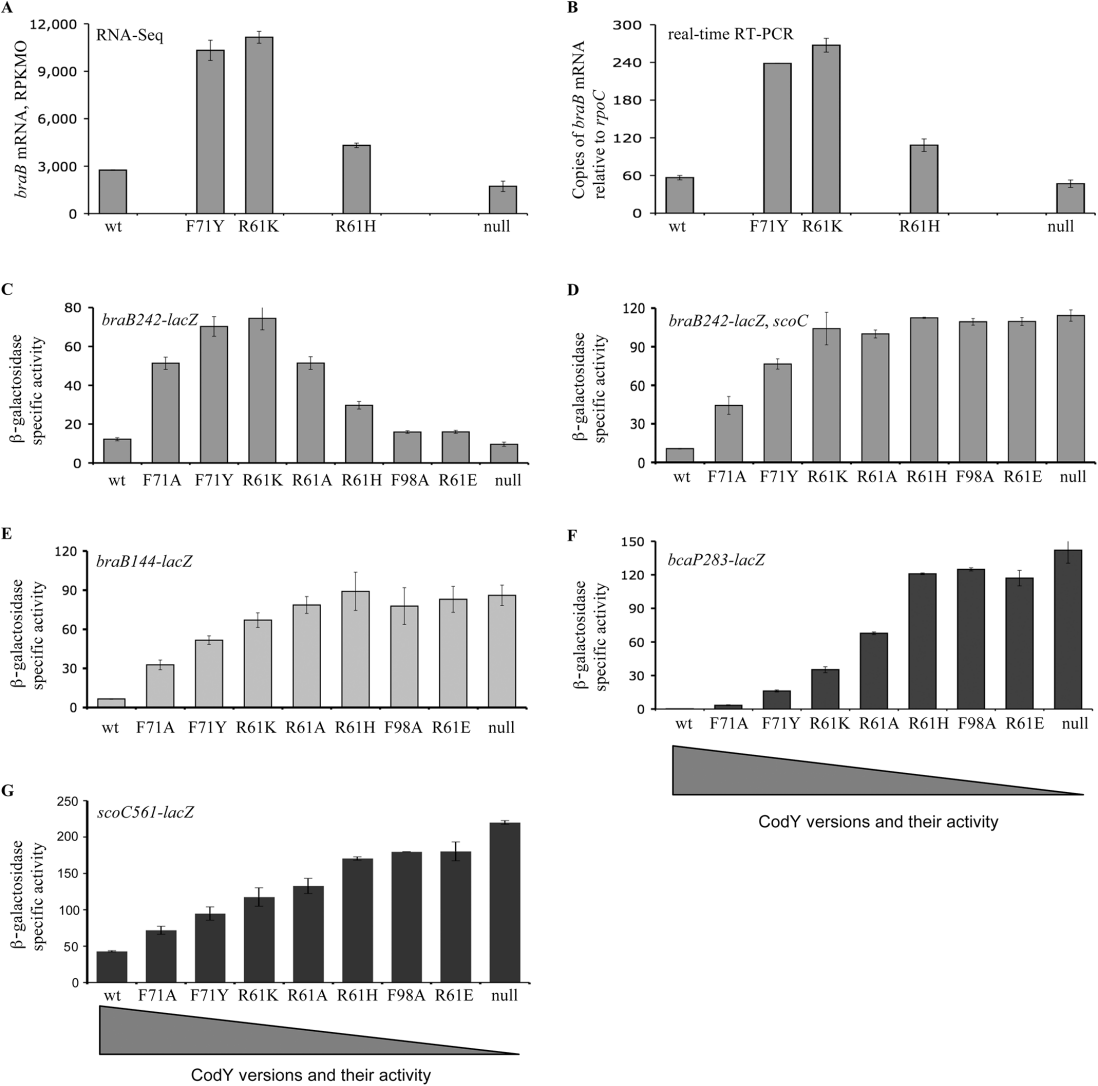
Expression of *braB*, *bcaP*, or *scoC* in mutants containing partially active versions of CodY. A and B. *braB* transcript abundance as determined by RNA-Seq (A) or quantitative, real-time RT-PCR (B). Cells were grown in TSS + 16 aa medium. The RNA-Seq values, expressed as reads per kilobase per million ORF (RPKMO) values, were taken from the Dataset S1 of Ref. [8]. The real-time RT-PCR results are expressed as *braB* transcript abundance relative to *rpoC* transcript abundance. Point mutant positions along the x-axis are arbitrary and do not imply a linear relationship. Data points are the means of at least two independent experiments, and the error bars show standard errors of the mean. C—G. Expression of *braB-lacZ*, *bcaP-lacZ*, and *scoC-lacZ* fusions in *scoC* ^*+*^ or *scoC* null mutant cells containing partially active versions of CodY. Cells were grown in TSS + 16 aa medium. β-Galactosidase activity was assayed and expressed in Miller units.

The analysis of Dataset S1 of Ref. (8) indicates that expression of *braB* determined by RNA-Seq experiments was up to 4.1-fold higher in three strains containing partially active versions of CodY, F71Y, R61K, or R61H (Fig 5A). The results of the RNA-Seq experiments were confirmed and extended by real-time RT-PCR and by analyzing expression of the *braB242-lacZ* transcriptional fusion in a larger collection of partial *codY* mutants (Fig 5B and 5C). The up-and-down expression pattern of the *braB* fusion, in which maximal activity was seen in mutants with intermediate levels of CodY activity, was in drastic contrast to the plateau-reaching expression pattern of the previously characterized CodY-repressed *bcaP283-lacZ* fusion (Fig 5F) [2] and all other CodY-regulated genes [2].

**Fig 6:**
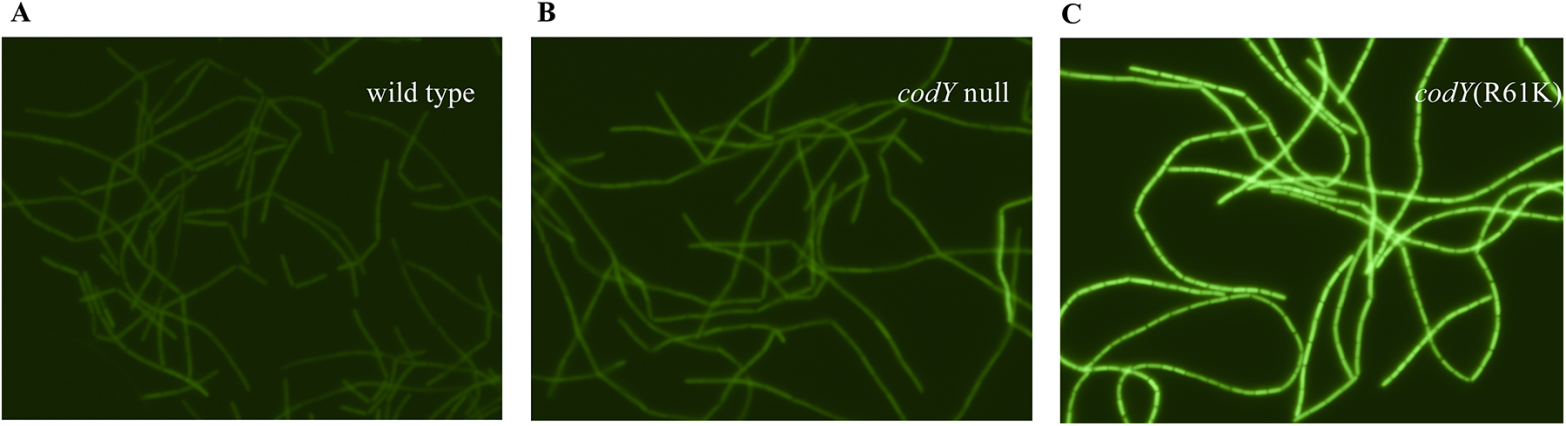
Expression of the *braB242-gfp* fusion in individual cells. Cells were grown in TSS + 16 aa medium. GFP fluorescence was detected using Zeiss Axio Observer.Z1 microscope. A. Strain BB4082 (wild type). B. Strain BB4083 (*codY*::*spc*). C. Strain BB4084 [*codY*(R61K)].

A *braB242-gfp* translational fusion was introduced into the wild-type strain, the *codY* null mutant, and a *codY* point mutant (R61K) strain with intermediate residual activity. In all cases, the level of *braB* expression was rather similar across the cell population (Fig 6), eliminating the possibility that a bistable expression pattern could explain our results. As expected, the *codY* (R61K) mutant strain had elevated expression compared to the wild-type and *codY* null mutant strains.

### Deletion analysis of the *braB* regulatory region

We initially hypothesized that the very unusual pattern of *braB* regulation observed results from CodY binding independently to negative and positive regulatory sites within the *braB* regulatory region. If so, the positive and negative effects of fully active CodY might balance each other, but, at intermediate levels of CodY activity, positive regulation might dominate. The CodY-binding sites I and II are located upstream and downstream of the *braB* promoter in positions appropriate for positive and negative regulation, respectively. To determine their independent effects, we created additional *lacZ* fusions containing truncated versions of the *braB* regulatory region lacking the upstream CodY-binding site III (*braB184-lacZ* and *braB162-lacZ)* or sites III and I (*braB144-lacZ*) or the downstream site II (*braB156-lacZ*) (Fig 1B). (Note that the *braB242-*, *braB184-*, *braB162-* and *braB144-lacZ* fusions have the identical junction with *lacZ*; their levels of activity can be directly compared. However, other fusions, such as *braB156-lacZ*, have different junctions; their activities in wild-type cells can only be compared to the activity of the same fusion in mutant strains or other fusions with a similar junction.)

Surprisingly, deletion of the upstream binding sites III and I did not cause any significant decrease in *braB* expression in wild-type cells (Table 2; compare strains BB3076, BB3719, BB3811, and BB3122), implying that these are not sites of positive regulation. On the other hand, the *braB162-lacZ* and *braB144-lacZ* fusions, but not the *braB184-lacZ* fusion, were derepressed 12-fold when *codY* was inactivated (Table 2), suggesting that *braB* expression is subject to negative regulation by CodY bound to the remaining downstream site II. If so, this regulation must be masked in other fusions by the action of a second repressor that binds to the sequence located between the 5’ ends of *braB184-lacZ* and *braB162-lacZ*. Interestingly, no up-and-down expression pattern in mutants with different levels of CodY activity was observed for the *braB144-lacZ* fusion, which lacks the putative binding site for the predicted second regulator (Fig 5E), suggesting that the latter is responsible for the unusual regulation. As expected from this new model, the *braB156-lacZ* fusion, which lacks the downstream CodY-binding site II, was not subject to regulation by CodY (Table 2).

### Mutations in the *braB* CodY-binding sites

To confirm that site I is not involved in *braB* regulation and to quantify more directly the contribution of site II, we changed the very highly conserved G9 and A10 residues of CodY-binding motifs 1, 2, and 3 to CC (the p1, p2, and p3 mutations, respectively) (Fig 1 and Table 1).

The p1 mutation in site I reduced ~10-fold the affinity of CodY for a fragment containing sites III and I, indicating that site I is the major contributor for CodY binding to this fragment (Fig 4B and 4C). However, as expected from our deletion analysis, the p1 mutation did not affect expression of the *braB242-lacZ* fusion (Table 3, strains BB3731 and BB3076). Thus, as noted previously, many CodY-binding sites have no physiological significance either because they are not positioned appropriately for regulation or because binding is too weak [7].

**Table 3:** Effect of mutations in the CodY-binding sites on *braB* expression.

Strain	Fusion type	Relevant genotype	β-Galactosidase activity	*codY/codY* ^*+*^ fold regulation	*scoC/scoC* ^*+*^ fold regulation
BB3076	*braB242p* ^*+*^	wild type	12.2±0.80	0.79	0.88
BB3079		*codY*	9.62±1.06		11.9
BB3847		*scoC*	10.7±0.18	10.7	
BB3835		*codY scoC*	114.2±4.40		
BB3731	*braB242p1*	wild type	14.2±0.20	1.1	0.91
BB3737		*codY*	15.3±1.50		7.7
BB3868		*scoC*	12.9±0.38	9.1	
BB3871		*codY scoC*	117.8±6.35		
BB3730	*braB242p2*	wild type	16.0±0.30	2.2	1.1
BB3736		*codY*	35.7±0.85		4.5
BB3867		*scoC*	17.3±1.55	9.2	
BB3870		*codY scoC*	159.4±0.20		
BB3729	*braB242p3*	wild type	97.3±1.60	0.13	1.4
BB3735		*codY*	12.5±1.35		5.5
BB3855		*scoC*	133.7±10.6	0.52	
BB3856		*codY scoC*	68.9±1.20		

Cells were grown and β-galactosidase activity was assayed as described in Table 2.

The p3 mutation reduced the affinity of CodY for site II ≥10-fold (Fig 4E). The p2 mutation did not affect binding of CodY to site II, but further decreased the ability of CodY to interact with this site if it already contained the p3 mutation (Fig 4F and 4G). Footprinting experiments showed that each mutation affected CodY binding to the region of site II, which corresponded to the respective motif (Fig 3B). Taking together the gel-shift and footprinting results, we conclude that interaction of CodY with motif 2 is weaker than with motif 3 and is partly dependent on simultaneous interaction of CodY with motif 3 (see below for the effect of p2 on *braB* regulation).

The p3 mutation increased expression of the *braB242-lacZ* fusion 8-fold consistent with relief from CodY-mediated repression (Table 3, strains BB3729 and BB3076). However, expression of the *braB242p3-lacZ* fusion was substantially reduced in a *codY* null mutant strain apparently due to repression by the second regulator (Table 3, strain BB3735). This result suggests strongly that the second regulator is active in *codY* mutant cells, but not in wild-type cells, i.e., its activity or expression is under negative CodY control. Paradoxically, this indicates that our initial hypothesis that *braB* regulation is subject to simultaneous positive and negative regulation by CodY was likely to be correct, though positive regulation appears to be indirect and mediated through regulation of the second repressor.

### Identification of ScoC as a second repressor of *braB*

CodY is known to regulate the expression of a small number of other regulatory proteins, including ScoC [6–8, 19, 29, 30]. ScoC is a repressor of multiple genes, including those encoding extracellular proteases and oligopeptide permeases, and is also involved in the regulation of sporulation [15–19, 31–33]. Though microarray experiments did not identify *braB* as a ScoC target [15], we decided to test whether ScoC is the second regulator of *braB* expression. No effect of a single *scoC* null mutation on expression of the *braB242-lacZ* fusion in TSS + 16 aa was detected (Table 2, strain BB3847). However, in a double *codY scoC* null mutant, expression of the fusion was 11- to 12-fold higher than in the wild-type strain or in *scoC* or *codY* single mutants (Table 2, strain BB3835), indicating that both CodY and ScoC contribute to negative regulation of *braB* but these effects cannot be dissected if either one of the regulators is active.

Expression of the same fusion in a double null mutant in TSS + 13 aa medium was very similar (Table 2), indicating that our original observation of higher *braB* expression under these growth conditions in a wild-type strain was indeed due to reduced CodY activity and its effect on ScoC expression.

As expected, in the absence of ScoC, the up-and-down expression pattern of the *braB242-lacZ* fusion in strains with different CodY activity was replaced by a plateau-reaching pattern, resembling that of the *bcaP283-lacZ* fusion, which is not subject to ScoC-mediated regulation (Fig 5D and 5F).

Expression of the *scoC561-lacZ* fusion in strains with different CodY activity also followed a plateau-reaching pattern, characteristic for most genes regulated by CodY, and did not correlate with expression from the *braB* promoter (Fig 5G).

### ScoC binding to the *braB* regulatory region

**Fig 7:**
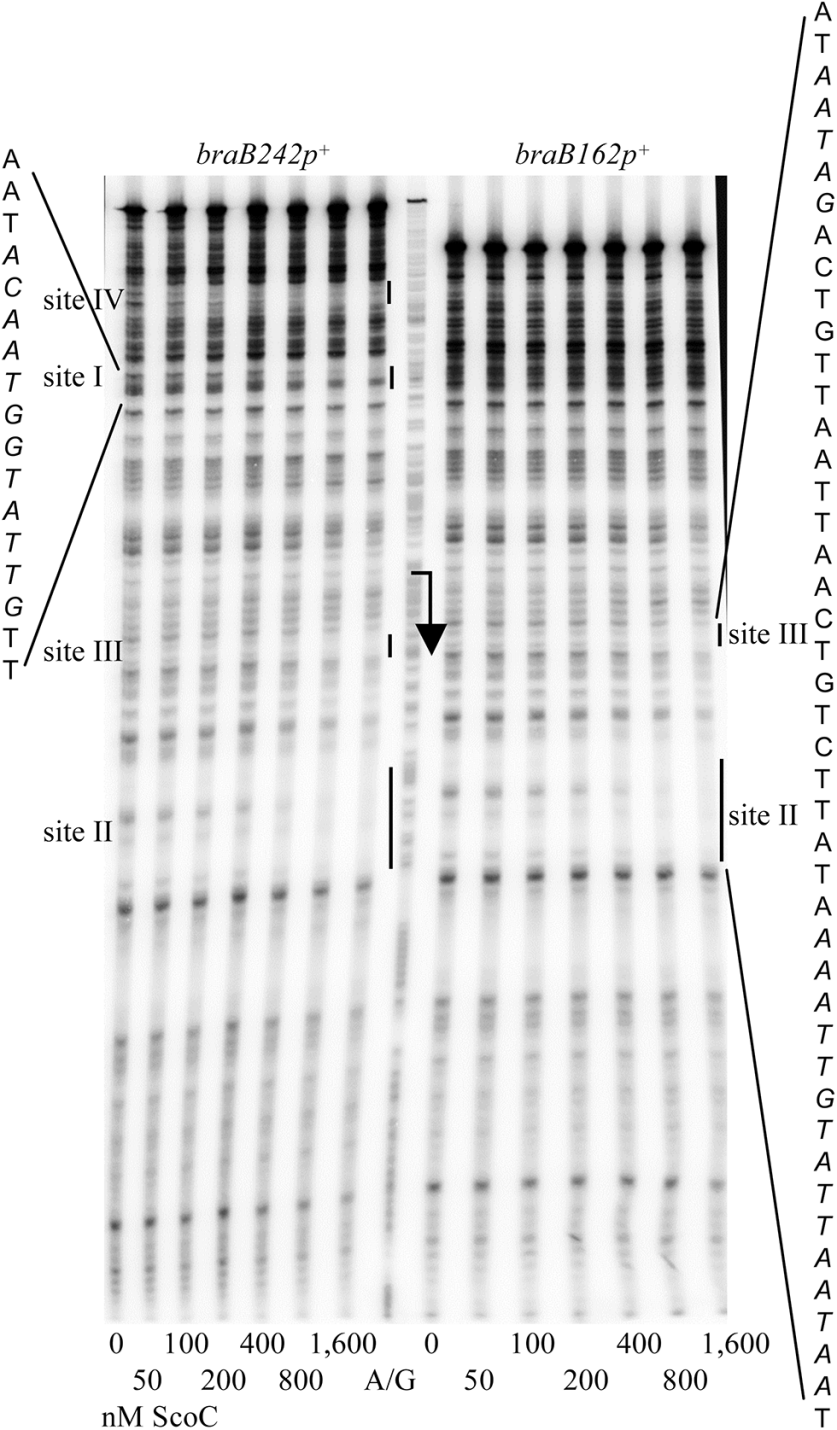
Determination of *braB* ScoC-binding regions. DNase I footprinting analysis of ScoC binding to the *braB* regulatory region. The *braB242p* ^*+*^ or *braB162p* ^*+*^ DNA fragment obtained by PCR with oligonucleotides oBB67 and oBB102 and labelled on the template strand was incubated with increasing amounts of purified ScoC and then with DNase I. See the legend to Fig 2B for additional details.

In DNase I footprinting experiments, ScoC protected two sites, I and II, within the *iscSB*-*braB* intergenic region from positions -79 to -68 and +43 to +57 of the template DNA strand with respect to the *braB* transcription start point, respectively (Figs 1A and 7). A short, weakly protected region, site III (possibly a part of site II), was also detected from positions +16 to +20. Binding of ScoC to the downstream sites II and III was independent of the presence of the upstream site I on the same DNA fragment (Fig 7).

**Fig 8:**
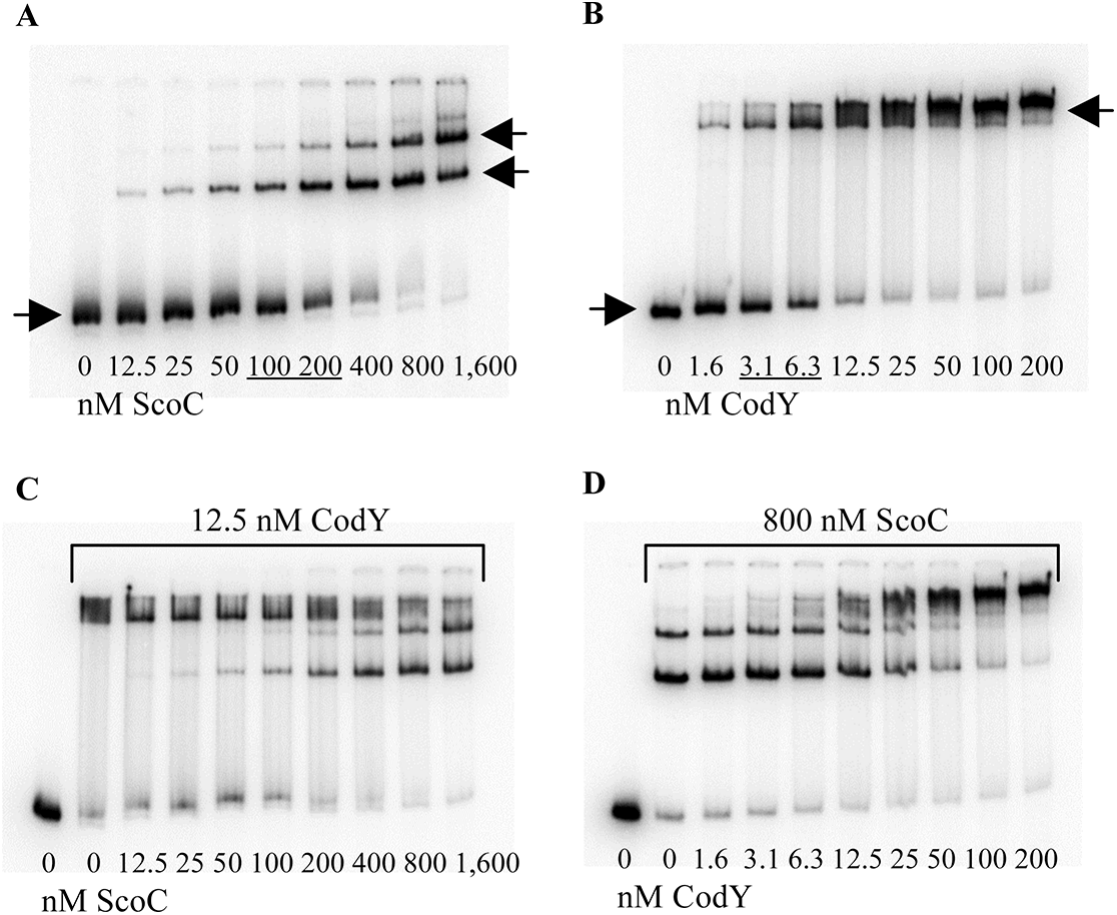
Competition between CodY and ScoC for *braB* binding. A and B. The *braB64* DNA fragment, obtained by PCR with oligonucleotides oBB730 and oBB731 and labelled on the template strand, was incubated for 32 min with increasing amounts of purified ScoC (A) or CodY (B) in the presence of 10 mM ILV. C and D. The *braB64* DNA fragment was preincubated for 16 min with 12.5 nM CodY (C) or 800 nM ScoC (D), and then increasing concentrations of either ScoC (C) or CodY (D) were added for an additional 16 min. The positions of the free DNA fragment and protein-DNA complexes are indicated by right-pointing and left-pointing arrows, respectively. Protein concentrations used (nM of monomer) are reported below each lane; concentrations corresponding to the apparent K_D_ for binding are underlined.

The downstream CodY- and ScoC-binding sites partly overlap (Fig 1A). To address the possibility that CodY and ScoC compete for binding at this location, we analyzed interaction of these proteins with a short, 64-bp *braB* fragment, containing CodY-binding site II and ScoC-binding sites II and III (Fig 1B). In accord with the results described above, ScoC bound this fragment in gel shift experiments less efficiently (K_D_≈150 nM) than did CodY (K_D_≈5 nM) (Fig 8A and 8B). Nevertheless, ScoC, in a concentration-dependent manner, was able to replace CodY efficiently in a preformed *braB*-CodY complex as evidenced by formation of ScoC-specific complexes with higher mobility and the decrease in the amount of *braB*-CodY complexes with lower mobility (Fig 8C). The CodY-mediated displacement of ScoC from the preformed *braB*-ScoC complex cannot be recognized confidently because of the low mobility of CodY-specific complexes (complexes containing both proteins would have a similar low mobility). However, by comparing and Fig 8B and 8D, it is clear that CodY bound much less efficiently to preformed *braB*-ScoC complexes than to free *braB* DNA, confirming competition between the two proteins for binding. A similar competition between CodY and ScoC was previously detected at the *oppA* promoter [19].

Another ScoC-binding site, site IV, was detected further upstream within the divergent *iscSB* gene (Figs 1A and 7). This site was not present in the *braB184-lacZ* fusion and therefore was not involved in the regulation described. No consensus ScoC-binding motifs, AATAnTATT [18], with ≤2 mismatches were detected within any of the *braB* binding sites.

### Deletion and mutational analysis of the role of ScoC-binding sites in *braB* expression

The locations of ScoC-binding sites I and II (Figs 1 and 7) correspond well to the binding sites for the predicted second regulator of *braB* determined by deletion analysis (Table 2). That is, expression of the *braB162-lacZ* and *braB144-lacZ* fusions, which lack the upstream ScoC-binding sites, was not affected by a *scoC* mutation even if the latter was present together with a *codY* mutation (Table 2). On the other hand, expression of the slightly longer *braB184-lacZ* fusion, which includes an intact ScoC-binding site I, as well as the downstream site II, was subject to full ScoC repression (as revealed in a double *codY scoC* mutant) (Table 2).

Expression of the *braB156-lacZ* and *braB181-lacZ* fusions, which carry the upstream ScoC binding site but lack the downstream site II, was also not affected by a *scoC* mutation (Table 2). A requirement for interaction with two (or more) binding sites within the same regulatory region appears to be a common theme for ScoC-mediated repression [18, 19, 34–36].

The lack of both ScoC- and CodY-mediated regulation explains why the *braB76-lacZ*, *braB156-lacZ*, and *braB181-lacZ* fusions are expressed at the same level in wild-type cells and in *codY* null mutant cells (Table 2). On the other hand, the *braB242p3-lacZ* fusion, which lost direct CodY-mediated regulation, is still subject to repression by increased levels of ScoC accumulated in a *codY* null mutant strain (Table 3).

Interestingly, the p2 mutation, designed to reduce binding of CodY to motif 2 of site II, in fact may affect ScoC interaction with the *braB* regulatory region. Indeed, the p2 mutation did not affect expression of the *braB242-lacZ* fusion in a wild-type strain, in which *scoC* is repressed, but did so in *codY* null mutant cells, in which ScoC is expressed (Table 3, strains BB3730 and BB3736); the p2 mutation is located 1 bp downstream of ScoC-binding site III (Fig 1).

The expression levels of different fusions and locations of the ScoC-binding sites confirmed that ScoC is the predicted second repressor of *braB*. As noted above, deleting of one of the ScoC-binding sites resulted in a plateau-reaching expression pattern of the *braB144-lacZ* fusion in strains with different CodY activity (Fig 5E).

## Discussion

**Fig 9:**
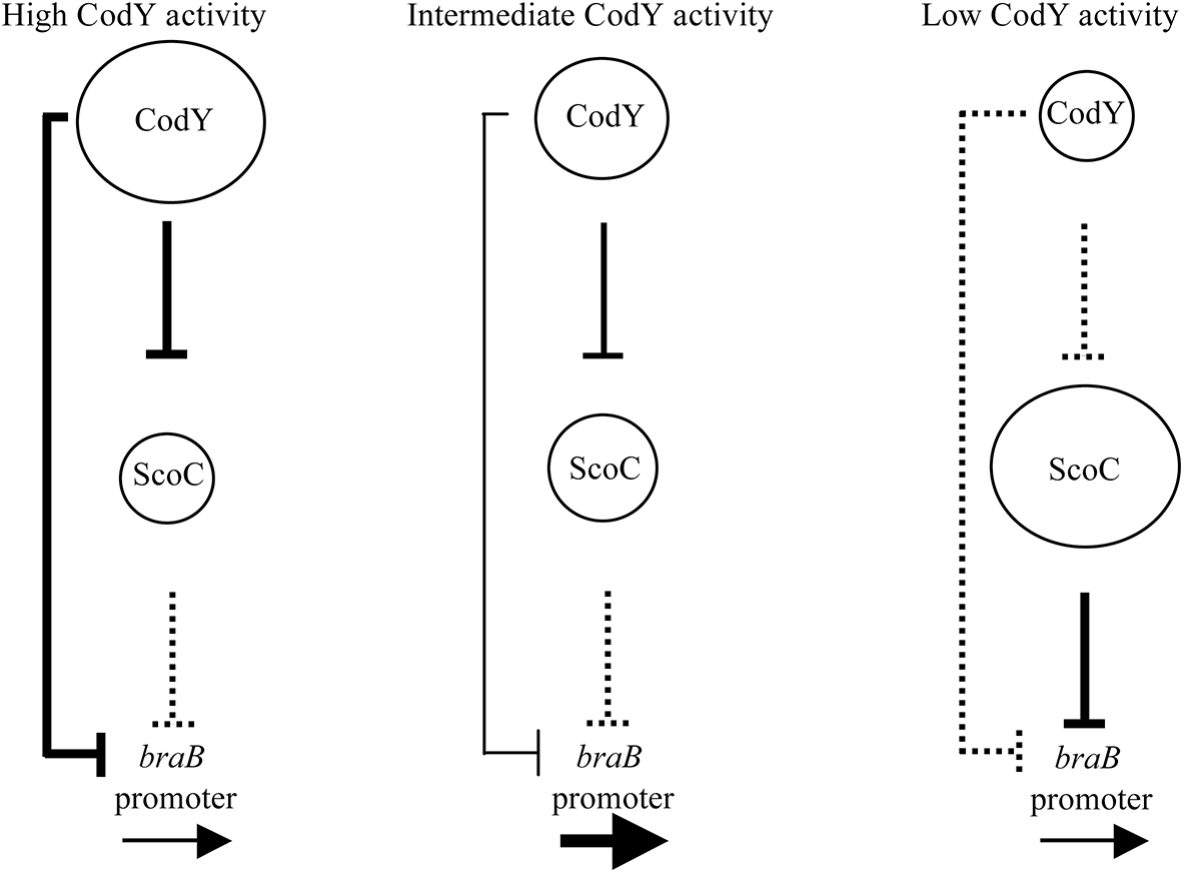
A model of regulation of the *braB* promoter by the combined actions of CodY and ScoC. The sizes of the circles reflect the relative amount of the active form of each protein. The solid vertical lines indicate relatively strong effects on transcription. Dotted lines indicate relatively weak effects on transcription. The boldness of the horizontal arrows indicates the relative strength of transcription of the target genes.

Although previous analysis did not detect any significant regulation of *braB* by CodY, we now know that *braB* is subject to complex CodY-mediated regulation by which the protein acts both as a direct repressor and as an indirect positive regulator. The positive effect of CodY is mediated by its repression of the gene encoding a second repressor of *braB*, ScoC. As a result, *braB* expression only escapes repression under conditions (e.g., during growth in a medium containing multiple amino acids but lacking ILV) in which CodY activity is limited enough to prevent repression of *braB*, but high enough to maintain sufficient repression of *scoC* (Fig 9).

Our previously described repression of *scoC* by CodY, coupled with ScoC autorepression [19], keeps the level of ScoC relatively low when cells are growing rapidly. Thus, CodY and ScoC are never fully active or inactive simultaneously. When CodY is inactive, the ScoC level is high enough to repress its target genes, including *braB*. When CodY is fully active, the ScoC level is insufficient for repression, but CodY is able to repress *braB* to the same level as fully active ScoC. Because we observe higher expression of *braB* under conditions of partial CodY activity, we suspect that as CodY activity declines, its binding to the *braB* regulatory region decreases more rapidly than does its binding to the *scoC* regulatory region. Alternatively, the affinity of ScoC for its *braB* binding site might be low enough that ScoC needs to reach a relatively high concentration in order to be effective; by the time this happens, CodY-mediated repression of *braB* is already very low. In addition, it is possible that the competition between more strongly binding CodY and more weakly binding ScoC for interaction with the same region of the *braB* regulatory region (at the downstream sites for each protein) may contribute to the differential response of *braB* expression to varying levels of CodY activity. As a result, even relatively small losses in activity of CodY, such as in CodY(F71A), allow neither efficient direct repression by CodY nor sufficient derepression of ScoC, which would compensate for the loss of CodY-mediated repression.

The novelty of *braB* regulation reinforces the view that important mechanisms of gene regulation can be missed by using regulatory protein null mutants as the only means of genetic analysis. A null mutant has no activity in any environment and at any stage of its life cycle, but in wild-type cells regulatory proteins are rarely, if ever, totally inactive. What normally varies is the fraction of the population of the regulator that is in the active state. Furthermore, interpreting the phenotype of a null mutant usually assumes that a regulatory protein is either only a positive regulator or only a negative regulator of its target gene(s). Studying the behavior of genes at different levels of a regulator’s activity has the potential to reveal more complex mechanisms in detail.

It should be noted that, although the complex pattern of *braB* regulation is very interesting, it is not common. Combined repression by CodY and ScoC has also been observed for the *B*. *subtilis opp* operon and *scoC* gene itself. However, in case of *opp*, ScoC-mediated repression was more efficient than CodY-mediated repression and was detected, although at a reduced level, even in *codY* ^*+*^ cells [19]. The opposite was true for expression of the *scoC* gene, whose regulation by CodY was detected even in *scoC* ^*+*^ cells [19]. Among CodY-regulated genes, only the *braB* gene has shown the described up-and-down pattern, i.e., expression was maximal at the intermediate levels of CodY activity [8]. It remains unknown whether additional regulatory inputs, e.g., through SalA-mediated regulation of *scoC* expression [37], affect interaction between ScoC and CodY.

We have recently characterized three permeases, BcaP, BraB, and BrnQ, involved in the BCAA uptake in *B*. *subtilis* cells [1]. The roles of different BCAA permeases in amino acid uptake under different growth conditions should reflect their levels of expression. The *bcaP (yhdG)* gene encodes the most efficient permease for isoleucine and valine and is one of the genes most highly repressed by CodY; expression of the *bcaP* gene is virtually abolished in amino acid-rich media [2, 6]. It is very likely that higher activity of BraB is not needed during strong nutrient limitation when CodY activity is very low, because BcaP is fully derepressed. It is also likely that when *bcaP* and *braB* are repressed by highly active CodY, the residual activity of BraB, together with BrnQ, is sufficient for the uptake of high concentrations of BCAA. However, the increase in BraB expression at partial activities of CodY may facilitate the uptake of intermediate concentrations of BCAA.

It is not uncommon for two regulators to control expression of the same gene in such a way that the lack of one regulator is fully compensated for by the increased activity of the other regulator and, as a result, no regulatory effect is observed in single null mutant strains. However, when such regulators act independently and do not form a feed-forward regulatory loop, the full compensatory effect should also be observed at intermediate activities of the regulator. The peculiarity of *braB* regulation is that the full compensatory effect of ScoC is seen only when CodY has very low or no activity.

The feed-forward regulatory loop formed by CodY and ScoC at the *braB* promoter, known as a type-2 incoherent loop, is an arrangement in which two regulatory proteins repress the same target gene and one of the regulators represses expression of the other [20, 21]. This regulatory mechanism may have evolved specifically to achieve higher expression of the *braB* gene at intermediate activities of CodY. Genes that are regulated by a single repressor are also expressed at a higher level when activity of the repressor is reduced. However, expression of such genes reaches its maximum only when the repressor is completely inactive; the regulatory mechanism of *braB* avoids this scenario.

## Materials and Methods

### Bacterial strains and culture media

The *B*. *subtilis* strains constructed and used in this study were all derivatives of strain SMY [38] and are described in Table 4 or in the text. *Escherichia coli* strain JM107 [39] was used for isolation of plasmids. Bacterial growth in DS nutrient broth or TSS 0.5% (w/v) glucose-0.2% (w/v) NH_4_Cl minimal medium was as described [2]. The TSS medium was supplemented as indicated with a mixture of 16 amino acids [40]. This mixture contained all amino acids commonly found in proteins (all concentrations in μg/ml) except for glutamine, asparagine, histidine, and tyrosine: glutamate-Na, 800; aspartate-K, 665; serine, 525; alanine, 445; arginine-HCl, 400; glycine, 375; isoleucine, leucine, and valine, 200 each; methionine, 160; tryptophan, 150; proline, threonine, phenylalanine, and lysine, 100 each; cysteine, 40. In some experiments, ILV were omitted from the amino acid-containing medium.

**Table 4:** *B*. *subtilis* strains used.

Strain	Genotype	Source or reference^a^
JH14272	*ΔamyE*::*[aph* Φ*(opp-lacZ)] ΔscoC*::*cat trpC2 pheA1*	[31]
SG81	*lacA*::*neo trpC2*	[42]
SRB465	*codY*(R61H*) ΔflgB2*::*erm*	[8]
SRB468	*codY*(F71Y*) ΔflgB2*::*erm*	[8]
BB1043	*codY*::*(erm*::*spc)*	[47]
BB2261	*lacA*::*(neo*::*spc) trpC2*	SG81xpVK71 [48]
BB2263	*lacA*::*(neo*::*spc)*	SMYxDNA(BB2261)
BB2511	Δ*amyE*::*spc lacA*::*tet*	[24]
BB2833	*codY*(R61A) *ΔamyE*::*spc lacA*::*tet*	[2]
BB2834	*codY*(R61K) *ΔamyE*::*spc lacA*::*tet*	[2]
BB2835	*codY*(R61E) *ΔamyE*::*spc lacA*::*tet*	[2]
BB2836	*codY*(F71A) *ΔamyE*::*spc lacA*::*tet*	[2]
BB2839	*codY*(F98A) *ΔamyE*::*spc lacA*::*tet*	[2]
BB3076	Δ*amyE*::[*erm* Φ(*braB242-lacZ*)] *lacA*::*tet*	BB2511xpBB1593
BB3122	Δ*amyE*::[*erm* Φ(*braB144-lacZ*)] *lacA*::*tet*	BB2511xpBB1596
BB3123	Δ*amyE*::[*erm* Φ(*braB156-lacZ*)] *lacA*::*tet*	BB2511xpBB1597
BB3714	*codY*(F71Y) *ΔflgB2*::*(erm*::*aphA3)*	SRB468xpBB1560
BB3716	*codY*(R61H*) ΔflgB2*::*(erm*::*aphA3)*	SRB465xpBB1560
BB3719	Δ*amyE*::[*erm* Φ(*braB184-lacZ*)] *lacA*::*tet*	BB2511xpBB1772
BB3729	Δ*amyE*::[*erm* Φ(*braB242p3-lacZ*)] *lacA*::*tet*	BB2511xpBB1773
BB3730	Δ*amyE*::[*erm* Φ(*braB242p2-lacZ*)] *lacA*::*tet*	BB2511xpBB1774
BB3731	Δ*amyE*::[*erm* Φ(*braB242p1-lacZ*)] *lacA*::*tet*	BB2511xpBB1775
BB3732	Δ*amyE*::[*erm* Φ(*braB242p2/p3-lacZ*)] *lacA*::*tet*	BB2511xpBB1776
BB3809	Δ*amyE*::[*erm* Φ(*braB242p1/p3-lacZ*)] *lacA*::*tet*	BB2511xpBB1801
BB3810	Δ*amyE*::[*erm* Φ(*braB242p1/p2-lacZ*)] *lacA*::*tet*	BB2511xpBB1802
BB3811	Δ*amyE*::[*erm* Φ(*braB162-lacZ*)] *lacA*::*tet*	BB2511xpBB1803
BB3821	Δ*amyE*::[*erm* Φ(*braB76-lacZ*)] *lacA*::*tet*	BB2511xpBB1804
BB3827	Δ*amyE*::[*erm* Φ(*braB181-lacZ*)] *lacA*::*tet*	BB2511xpBB1807
BB3828	Δ*amyE*::[*erm* Φ(*braB101-lacZ*)] *lacA*::*tet*	BB2511xpBB1808
BB4082	Δ*lacA*::[*tet* Φ*(braB-gfp)*]	BB2263xpBB1845

^a^The symbol × indicates transformation by plasmid or chromosomal DNA

### DNA manipulations

Methods for common DNA manipulations, transformation, primer extension, and sequence analysis were as previously described [24, 41]. All oligonucleotides used in this work are described in Table 5. Chromosomal DNA of *B*. *subtilis* strain SMY or plasmids constructed in this work were used as templates for PCR. All cloned PCR-generated fragments were verified by sequencing.

**Table 5:** Oligonucleotides used.*^a^*

Name	Sequence^a^	Specificity
Flanking forward primers		
oBB67	5’- GCTTCTAAGTCTTATTTCC	*erm* (pHK23)
oBB358	5’- CTGCAGGTCGACTCTAG	pHK23
oBB417	5’- CTTCGTCTAGAACTATTATCTAAATATATC	*braB242*
oBB422	5’- GAAAATCTAGAAAAACTAGTATTGAC	*braB144*
oBB645	5’- ACAAGTCTAGATATTTATGTTACCATAAC	*braB184*
oBB724	5’- GCTTCGAATTCGAACTATTATCTAAATATATC	*braB242-gfp*
oBB730	5’- TCGTTTCGTCATATTATC	*braB64*
oSRB339	5’- TTAACCGGTAAGGACGCTAAAG	*braB*
oSRB194	5'- CCGTTCATGGTCTTTTGGTG	*rpoC*
Flanking reverse primers		
oBB102	5’- CACCTTTTCCCTATATAAAAGC	*lacZ* (pHK23)
oBB418	5’- AATGGAAGCTTTGACAGGCAGTGAG	*braB242*
oBB423	5’- CAGATAAGCTTACGAAACGAACAAATC	*braB156*
oBB688	5’- TAAAAAAGCTTGTCAATTAATTGTCAG	*braB181*
oBB725	5’- ATAATGTCGACTTTGACAGGCAGTG	*braB242-gfp*
oBB731	5’- AATCCTCCTAATAATTATGTT	*braB64*
oSRB340	5’- GTTCTCGGGATGGCGAATAA	*braB*
oSRB195	5'- TTTAGCCCGTGTTACTTCGAC	*rpoC*
Internal mutagenic forward primer		
oBB642	5’- CAATTAATTGACA**CC**ATATTTTAAC	*braB242p3*
oBB644	5’- CATATTATCT**CC**CAATTAATTGAC	*braB242p2*
oBB647	5’- CAAAATTTAATA**CC**AAATTACGAAAAAC	*braB242p1*
Internal mutagenic reverse primer		
oBB641	5’- GTTAAAATAT**GG**TGTCAATTAATTG	*braB242p3*
oBB643	5’- GTCAATTAATTG**GG**AGATAATATG	*braB242p2*
oBB646	5’- GTTTTTCGTAATTT**GG**TATTAAATTTTG	*braB242p1*

^***a***^The restriction sites are underlined. Substituted nucleotides creating mutations in the CodY-binding motifs are in boldface.

### Construction of transcriptional *braB-lacZ* fusions

Plasmid pBB1593 (*braB242-lacZ)* was created by cloning the XbaI- and HindIII-treated PCR product in an integrative plasmid pHK23 (*erm*) [24]. The 0.24-kb *braB* PCR product, containing the entire *braB* regulatory region, was synthesized with oBB417 and oBB418 as primers. Plasmids pBB1596 (*braB144-lacZ*) or pBB1772 (*braB184-lacZ*), containing the *braB* regulatory region truncated from the 5’ end, were constructed in a similar way using oBB422 or oBB645, respectively, instead of oBB417. Plasmids pBB1597 (*braB156-lacZ*) and pBB1807 (*braB181-lacZ*), containing the *braB* regulatory region truncated from the 3’ end, were created as pBB1593 but using oBB423 or oBB688, respectively, instead of oBB418. Plasmids pBB1803 (*braB162-lacZ*), pBB1804 (*braB76-lacZ*), and pBB1808 (*braB101-lacZ*), in which the *braB* regulatory region was additionally truncated at the 5’ end, were constructed as pBB1593, pBB1597, and pBB1807, respectively, but using the ApoI and HindIII-digested PCR products that were cloned in pHK23, treated with EcoRI and HindIII.

*B*. *subtilis* strains carrying various *lacZ* fusions at the *amyE* locus (Table 4) were isolated after transforming strain BB2511 (*amyE*::*spc lacA*) with the appropriate plasmids, by selecting for resistance to erythromycin, conferred by the plasmids, and screening for loss of the spectinomycin-resistance marker, which indicated a double crossover, homologous recombination event. Strain BB2511 and all its derivatives have very low endogenous β-galactosidase activity due to a null mutation in the *lacA* gene [42].

### Mutations in the CodY-binding sites

Plasmids pBB1773 (*braB242p3-lacZ*), pBB1774 (*braB242p2-lacZ*), and pBB1775 (*braB242p1-lacZ*), containing 2-bp substitution mutations in CodY-binding motifs, were constructed as described for pBB1593 using fragments generated by two-step overlapping PCR.

In the first step, a product containing the 5’ part of the *braB* regulatory region was synthesized by using oligonucleotide oBB417 as the forward primer and mutagenic oligonucleotide oBB641, or oBB643, or oBB646 as the reverse primer. A product containing the 3’ part of the *braB* regulatory region was synthesized by using mutagenic oligonucleotides oBB642, or oBB644, or oBB647 as the forward primer and oligonucleotide oBB418 as the reverse primer. The PCR products were used in a second, splicing step of PCR mutagenesis as overlapping templates to generate a modified fragment containing the entire *braB* regulatory region; oligonucleotides oBB417 and oBB418 served as the forward and reverse PCR primers, respectively.

Plasmid pBB1776 (*braB242p2/p3-lacZ*), pBB1801 (*braB242p1/p3-lacZ*), and pBB1802 (*braB242p1/p2-lacZ*), containing two mutations, each, were constructed in a similar way, but using a plasmid, containing one of the mutations, pBB1773 or pBB1774, as template for PCR.

Truncated plasmids, containing mutations in the *braB* regulatory region, were constructed in the same way as plasmids without mutations.

A conversion plasmid for replacing the *aphA3* marker for the *erm* marker, originating from Tn917, was constructed by cloning the 1.5-kb SmaI-StuI fragment of pDG782 [43] into the SnaBI site of pJPM8 [44]. In the resulting plasmid, pBB1560, the *aphA3* gene of pDG782, conferring resistance to kanamycin or neomycin, is flanked by 5’ and 3’ parts of the *erm* cassette of pJPM8; the orientation of the *aphA* gene coincides with that of *erm*.

### Labeling of DNA fragments

The PCR products containing the regulatory region of the *braB* gene were synthesized using *braB*-specific oliginucleotides or vector-specific oligonucleotides oBB67 or oBB358 and oBB102, as the forward and reverse primers, respectively. The reverse primer for each PCR reaction (which would prime synthesis of the template strand of the PCR product) was labeled using T4 polynucleotide kinase and [γ-32P]-ATP. oBB67 and oBB358 start 96 bp or 12 bp upstream of the XbaI site (and 112 bp or 28 bp upstream of the EcoRI site) used for cloning, respectively, and oBB102 starts 36 bp downstream of the HindIII site that serves as a junction between the promoters and the *lacZ* part of the *braB* fusion.

The procedures for gel shift and DNase I footprinting experiments were as described [19].

### Construction of a translational *braB-gfp* fusion and fluorescence microscopy

A 0.24-kb *braB* PCR product, containing the entire *braB* regulatory region, was synthesized with oBB724 and oBB725 as primers. Plasmid pBB1845 (*braB242-gfp)* was created by cloning the EcoRI- and SalI-treated PCR product between the EcoRI and XhoI sites of an integrative plasmid pMMB759 (*tet*), containing a gene encoding a monomeric version (A206K) of GFPmut2 [45]. The *braB* insert within pBB1845 was identical to the insert in pBB1593 (*braB242-lacZ)*. *B*. *subtilis* strain BB4082 carrying the *braB242-gfp* fusion at the *lacA* locus was isolated after transforming strain BB2263 (*lacA*::*spc*) with pBB1845, by selecting for resistance to tetracycline, conferred by the plasmid, and screening for loss of the spectinomycin-resistance marker, which indicated a double crossover, homologous recombination event.

Cells, containing the *braB242-gfp* fusion, were grown until mid- to late-exponential phase in TSS + 16 aa medium, centrifuged and resuspended in TSS medium at OD_600_≈3. The images were collected at a 1,500 ms exposure time using the 100x (1.3 N.A.) objective of the Zeiss Axio Observer.Z1 fluorescent microscope (Zeiss) with the Colibri.2 LED light source, and the ORCA-R^2^ digital charge-coupled device camera (C10600, Hamamatsu). Zen Pro 2012 software (Zeiss) was used to acquire, view, and analyze the images.

### Protein purification

CodY-His_5_ and His_6_-ScoC were purified to near homogeneity as described previously [19, 24].

### Enzyme assays

β-Galactosidase specific activity was determined as described previously [46].

### Real-time RT-PCR

RNA isolation, DNA depletion, and cDNA synthesis were performed as previously described [14]. Quantitative, real-time RT-PCR was used to measure steady state *braB* transcript abundance during exponential growth using oligonucleotides oSRB339 and oSRB340 as described [14], except that we used *B*. *subtilis* strain SMY chromosomal DNA to generate the standard curve. *rpoC* transcript was used to normalize mRNA abundance.

## References

[1] Belitsky BR. Role of branched-chain amino acid transport in *Bacillus subtilis* CodY activity. J Bacteriol. 2015;197(8):1330–8. 10.1128/JB.02563-14 25645558 PMC4372739

[2] Belitsky BR, Sonenshein AL. Contributions of multiple binding sites and effector-independent binding to CodY-mediated regulation in *Bacillus subtilis* . J Bacteriol. 2011;193(2):473–84. 10.1128/JB.01151-10 21097623 PMC3019828

[3] Sonenshein AL. CodY, a global regulator of stationary phase and virulence in Gram-positive bacteria. Curr Opin Microbiol. 2005;8(2):203–7. 15802253 10.1016/j.mib.2005.01.001

[4] Sonenshein AL. Control of key metabolic intersections in *Bacillus subtilis* . Nat Rev Microbiol. 2007;5(12):917–27. 17982469 10.1038/nrmicro1772

[5] Belitsky BR, Gustafsson MC, Sonenshein AL, Von Wachenfeldt C. An *lrp*-like gene of *Bacillus subtilis* involved in branched-chain amino acid transport. J Bacteriol. 1997;179(17):5448–57. 9287000 10.1128/jb.179.17.5448-5457.1997 PMC179416

[6] Molle V, Nakaura Y, Shivers RP, Yamaguchi H, Losick R, Fujita Y, et al Additional targets of the *Bacillus subtilis* global regulator CodY identified by chromatin immunoprecipitation and genome-wide transcript analysis. J Bacteriol. 2003;185(6):1911–22. 12618455 10.1128/JB.185.6.1911-1922.2003 PMC150151

[7] Belitsky BR, Sonenshein AL. Genome-wide identification of *Bacillus subtilis* CodY-binding sites at single-nucleotide resolution. Proc Natl Acad Sci U S A. 2013;110(17):7026–31. 10.1073/pnas.1300428110 23569278 PMC3637721

[8] Brinsmade SR, Alexander EL, Livny J, Stettner AI, Segre D, Rhee KY, et al Hierarchical expression of genes controlled by the *Bacillus subtilis* global regulatory protein CodY. Proc Natl Acad Sci USA. 2014;111(22):8227–32. 10.1073/pnas.1321308111 24843172 PMC4050614

[9] Guedon E, Serror P, Ehrlich SD, Renault P, Delorme C. Pleiotropic transcriptional repressor CodY senses the intracellular pool of branched-chain amino acids in *Lactococcus lactis* . Mol Microbiol. 2001;40(5):1227–39. 11401725 10.1046/j.1365-2958.2001.02470.x

[10] Petranovic D, Guedon E, Sperandio B, Delorme C, Ehrlich D, Renault P. Intracellular effectors regulating the activity of the *Lactococcus lactis* CodY pleiotropic transcription regulator. Mol Microbiol. 2004;53(2):613–21. 15228538 10.1111/j.1365-2958.2004.04136.x

[11] Shivers RP, Sonenshein AL. Activation of the *Bacillus subtilis* global regulator CodY by direct interaction with branched-chain amino acids. Mol Microbiol. 2004;53(2):599–611. 15228537 10.1111/j.1365-2958.2004.04135.x

[12] Ratnayake-Lecamwasam M, Serror P, Wong KW, Sonenshein AL. *Bacillus subtilis* CodY represses early-stationary-phase genes by sensing GTP levels. Genes Dev. 2001;15(9):1093–103. 11331605 10.1101/gad.874201 PMC312684

[13] Handke LD, Shivers RP, Sonenshein AL. Interaction of *Bacillus subtilis* CodY with GTP. J Bacteriol. 2008;190(3):798–806. 17993518 10.1128/JB.01115-07 PMC2223590

[14] Brinsmade SR, Sonenshein AL. Dissecting complex metabolic integration provides direct genetic evidence for CodY activation by guanine nucleotides. J Bacteriol. 2011;193(20):5637–48. 10.1128/JB.05510-11 21856856 PMC3187194

[15] Caldwell R, Sapolsky R, Weyler W, Maile RR, Causey SC, Ferrari E. Correlation between *Bacillus subtilis scoC* phenotype and gene expression determined using microarrays for transcriptome analysis. J Bacteriol. 2001;183(24):7329–40. 11717292 10.1128/JB.183.24.7329-7340.2001 PMC95582

[16] Dod B, Balassa G, Raulet E, Jeannoda V. Spore control (*sco*) mutations in *Bacillus subtilis*. II. Sporulation and the production of extracellular protease and α-amylase by *scoC* mutants. Molec Gen Genet. 1978;163:45–56.

[17] Higerd TB, Hoch JA, Spizizen J. Hyperprotease-producing mutants of *Bacillus subtilis* . J Bacteriol. 1972;112(2):1026–8. 4628743 10.1128/jb.112.2.1026-1028.1972 PMC251520

[18] Kallio PT, Fagelson JE, Hoch JA, Strauch MA. The transition state regulator Hpr of *Bacillus subtilis* is a DNA-binding protein. J Biol Chem. 1991;266(20):13411–7. 1906467

[19] Belitsky BR, Barbieri G, Albertini AM, Ferrari E, Strauch MA, Sonenshein AL. Interactive Regulation by the *Bacillus subtilis* Global Regulators CodY and ScoC. Mol Microbiol. 2015;97(4):698–716. 10.1111/mmi.13056 25966844 PMC4550321

[20] Mangan S, Alon U. Structure and function of the feed-forward loop network motif. Proc Natl Acad Sci USA. 2003;100(21):11980–5. 14530388 10.1073/pnas.2133841100 PMC218699

[21] Alon U. Network motifs: theory and experimental approaches. Nat Rev Genet. 2007;8(6):450–61. 17510665 10.1038/nrg2102

[22] den Hengst CD, van Hijum SA, Geurts JM, Nauta A, Kok J, Kuipers OP. The *Lactococcus lactis* CodY regulon: identification of a conserved cis-regulatory element. J Biol Chem. 2005;280(40):34332–42. 16040604 10.1074/jbc.M502349200

[23] Guedon E, Sperandio B, Pons N, Ehrlich SD, Renault P. Overall control of nitrogen metabolism in *Lactococcus lactis* by CodY, and possible models for CodY regulation in Firmicutes. Microbiology. 2005;151(Pt 12):3895–909. 16339935 10.1099/mic.0.28186-0

[24] Belitsky BR, Sonenshein AL. Genetic and biochemical analysis of CodY-binding sites in *Bacillus subtilis* . J Bacteriol. 2008;190(4):1224–36. 18083814 10.1128/JB.01780-07 PMC2238193

[25] Belitsky BR, Sonenshein AL. Roadblock repression of transcription by *Bacillus subtilis* CodY. J Mol Biol. 2011;411(4):729–43. 10.1016/j.jmb.2011.06.012 21699902 PMC3156270

[26] Belitsky BR, Sonenshein AL. CodY-mediated regulation of guanosine uptake in *Bacillus subtilis* . J Bacteriol. 2011;193(22):6276–87. 10.1128/JB.05899-11 21926227 PMC3209203

[27] Slack FJ, Mueller JP, Sonenshein AL. Mutations that relieve nutritional repression of the *Bacillus subtilis* dipeptide permease operon. J Bacteriol. 1993;175(15):4605–14. 8335620 10.1128/jb.175.15.4605-4614.1993 PMC204911

[28] Villapakkam AC, Handke LD, Belitsky BR, Levdikov VM, Wilkinson AJ, Sonenshein AL. Genetic and biochemical analysis of the interaction of *Bacillus subtilis* CodY with branched-chain amino acids. J Bacteriol. 2009;191(22):6865–76. 10.1128/JB.00818-09 19749041 PMC2772489

[29] Serror P, Sonenshein AL. CodY is required for nutritional repression of *Bacillus subtilis* genetic competence. J Bacteriol. 1996;178(20):5910–5. 8830686 10.1128/jb.178.20.5910-5915.1996 PMC178446

[30] Smits WK, Hoa TT, Hamoen LW, Kuipers OP, Dubnau D. Antirepression as a second mechanism of transcriptional activation by a minor groove binding protein. Mol Microbiol. 2007;64(2):368–81. 17493123 10.1111/j.1365-2958.2007.05662.x PMC3831528

[31] Koide A, Perego M, Hoch JA. ScoC regulates peptide transport and sporulation initiation in *Bacillus subtilis* . J Bacteriol. 1999;181(13):4114–7. 10383984 10.1128/jb.181.13.4114-4117.1999 PMC93906

[32] Dod B, Balassa G. Spore control (*sco*) mutations in *Bacillus subtilis*. III. Regulation of extracellular protease synthesis in the spore control mutations *scoC* . Molec Gen Genet. 1978;163:57–63.

[33] Perego M, Hoch JA. Sequence analysis and regulation of the *hpr* locus, a regulatory gene for protease production and sporulation in *Bacillus subtilis* . J Bacteriol. 1988;170(6):2560–7. 3131303 10.1128/jb.170.6.2560-2567.1988 PMC211172

[34] Henner DJ, Ferrari E, Perego M, Hoch JA. Location of the targets of the *hpr-97*, *sacU32*(Hy), and *sacQ36*(Hy) mutations in upstream regions of the subtilisin promoter. J Bacteriol. 1988;170(1):296–300. 2447063 10.1128/jb.170.1.296-300.1988 PMC210641

[35] Ogura M, Matsuzawa A, Yoshikawa H, Tanaka T. *Bacillus subtilis* SalA (YbaL) negatively regulates expression of *scoC*, which encodes the repressor for the alkaline exoprotease gene, *aprE* . J Bacteriol. 2004;186(10):3056–64. 15126467 10.1128/JB.186.10.3056-3064.2004 PMC400612

[36] Toma S, Del Bue M, Pirola A, Grandi G. *nprR1* and *nprR2* regulatory regions for neutral protease expression in *Bacillus subtilis* . J Bacteriol. 1986;167(2):740–3. 3090022 10.1128/jb.167.2.740-743.1986 PMC212956

[37] Derouiche A, Shi L, Bidnenko V, Ventroux M, Pigonneau N, Franz-Wachtel M, et al *Bacillus subtilis* SalA is a phosphorylation-dependent transcription regulator that represses *scoC* and activates the production of the exoprotease AprE. Mol Microbiol. 2015;97(6):1195–208. 10.1111/mmi.13098 26094643

[38] Zeigler DR, Pragai Z, Rodriguez S, Chevreux B, Muffler A, Albert T, et al The origins of 168, W23, and other *Bacillus subtilis* legacy strains. J Bacteriol. 2008;190(21):6983–95. 10.1128/JB.00722-08 18723616 PMC2580678

[39] Yanisch-Perron C, Vieira J, Messing J. Improved M13 phage cloning vectors and host strains: nucleotide sequences of the M13mp18 and pUC19 vectors. Gene. 1985;33(1):103–19. 2985470 10.1016/0378-1119(85)90120-9

[40] Atkinson MR, Wray LV Jr., Fisher SH. Regulation of histidine and proline degradation enzymes by amino acid availability in *Bacillus subtilis* . J Bacteriol. 1990;172(9):4758–65. 2118500 10.1128/jb.172.9.4758-4765.1990 PMC213128

[41] Sambrook J, Fritsch EF, Maniatis TJ. Molecular cloning: a laboratory manual, 2nd ed Cold Spring Harbor Laboratory, Cold Spring Harbor, NY 1989.

[42] Daniel RA, Haiech J, Denizot F, Errington J. Isolation and characterization of the *lacA* gene encoding beta-galactosidase in *Bacillus subtilis* and a regulator gene, *lacR* . J Bacteriol. 1997;179(17):5636–8. 9287030 10.1128/jb.179.17.5636-5638.1997 PMC179446

[43] Guerout-Fleury AM, Shazand K, Frandsen N, Stragier P. Antibiotic-resistance cassettes for *Bacillus subtilis* . Gene. 1995;167(1–2):335–6. 8566804 10.1016/0378-1119(95)00652-4

[44] Mueller JP, Bukusoglu G, Sonenshein AL. Transcriptional regulation of *Bacillus subtilis* glucose starvation-inducible genes: control of *gsiA* by the ComP-ComA signal transduction system. J Bacteriol. 1992;174(13):4361–73. 1378051 10.1128/jb.174.13.4361-4373.1992 PMC206221

[45] Berkmen MB, Lee CA, Loveday EK, Grossman AD. Polar positioning of a conjugation protein from the integrative and conjugative element ICEBs1 of *Bacillus subtilis* . J Bacteriol. 2010;192(1):38–45. 10.1128/JB.00860-09 19734305 PMC2798270

[46] Belitsky BR, Sonenshein AL. Role and regulation of *Bacillus subtilis* glutamate dehydrogenase genes. J Bacteriol. 1998;180(23):6298–305. 9829940 10.1128/jb.180.23.6298-6305.1998 PMC107716

[47] Barbieri G, Voigt B, Albrecht D, Hecker M, Albertini AM, Sonenshein AL, et al CodY regulates expression of the *Bacillus subtilis* extracellular proteases Vpr and Mpr. J Bacteriol. 2015;197:1423–32. 10.1128/JB.02588-14 25666135 PMC4372738

[48] Chary VK, Amaya EI, Piggot PJ. Neomycin- and spectinomycin-resistance replacement vectors for *Bacillus subtilis* . FEMS Microbiol Lett. 1997;153(1):135–9. 9252583 10.1111/j.1574-6968.1997.tb10474.x

